# Full-Genome Characterization and Genetic Evolution of West African Isolates of Bagaza Virus

**DOI:** 10.3390/v10040193

**Published:** 2018-04-13

**Authors:** Martin Faye, Oumar Faye, Moussa Moise Diagne, Gamou Fall, Manfred Weidmann, Mbacke Sembene, Amadou Alpha Sall, Ousmane Faye

**Affiliations:** 1 Virology Pole, Institut Pasteur of Dakar, Dakar 220, Senegal; martin.faye@pasteur.sn (M.F.); Oumar.Faye@pasteur.sn (O.F.); MoussaMoise.DIAGNE@pasteur.sn (M.M.D.); gamou.fall@pasteur.sn (G.F.); Amadou.SALL@pasteur.sn (A.A.S.); 2 Faculty for Sciences and technics, Cheikh Anta Diop University, Dakar 5005, Senegal; mbacke.sembene@ucad.edu.sn; 3 Institute of Aquaculture University of Stirling, Stirling FK9 4LA, Scotland, UK; m.w.weidmann@stir.ac.uk

**Keywords:** Bagaza virus, West Africa, full-genome characterization, genetic evolution, codon adaptation

## Abstract

Bagaza virus is a mosquito-borne flavivirus, first isolated in 1966 in Central African Republic. It has currently been identified in mosquito pools collected in the field in West and Central Africa. Emergence in wild birds in Europe and serological evidence in encephalitis patients in India raise questions on its genetic evolution and the diversity of isolates circulating in Africa. To better understand genetic diversity and evolution of Bagaza virus, we describe the full-genome characterization of 11 West African isolates, sampled from 1988 to 2014. Parameters such as genetic distances, N-glycosylation patterns, recombination events, selective pressures, and its codon adaptation to human genes are assessed. Our study is noteworthy for the observation of N-glycosylation and recombination in Bagaza virus and provides insight into its Indian origin from the 13th century. Interestingly, evidence of Bagaza virus codon adaptation to human house-keeping genes is also observed to be higher than those of other flaviviruses well known in human infections. Genetic variations on genome of West African Bagaza virus could play an important role in generating diversity and may promote Bagaza virus adaptation to other vertebrates and become an important threat in human health.

## 1. Introduction

Bagaza virus (BAGV) belongs to the Flaviridae family, Flavivirus genus and Ntaya serological group. BAGV is a mosquito-transmitted virus, which was first isolated in the Bagaza district of the Central African Republic (CAR), in 1966, from a pool of mixed-species female Culex spp. mosquitoes during entomological investigations [1]. As is characteristic of flaviviruses, BAGV possesses a linear single-stranded, positive-sense RNA genome [2]. The BAGV genome is 10,941 nucleotides in length, encoding a single polyprotein (3426 amino acids) from which 11 viral proteins are derived, and flanked by 5′ and 3′ untranslated regions (UTRs) of 94 and 556 nt, respectively [3].

BAGV has been isolated repeatedly with a high titer from different species of mosquitoes in Central and West African countries [4,5,6], and in India, where serological investigations suggested subclinical infections in humans [7,8]. Despite this widespread circulation of BAGV, outbreaks involving humans or animals have not yet been reported from these countries. Subsequently, in September 2010, BAGV was associated with a high mortality among game birds (Partridges and pheasants) in southern Spain, the first detection of BAGV in Europe and the first isolation from a vertebrate host [9,10]. However, it is not surprising that BAGV infects birds since it has been shown to be synonymous with Israel turkey meningoencephalitis virus (ITV), a pathogen affecting poultry (turkeys) and close to West Nile virus (WNV) in several genomic regions [3,11]. Although diverse studies have contributed greatly to our understanding of the transmission modes of BAGV [8,12] and the pathogenesis of BAGV infection [13], only one study has addressed BAGV genetic diversity, reporting a high homology >90% between Central African Republic, Indian and Spanish BAGV isolates [9]. In West Africa, there is a lack of data on BAGV genetic diversity. To fill this gap and gain better insights into BAGV molecular evolution, we characterized the full-genome of 11 BAGV isolates, sampled from 1988 to 2014 in Senegal and Côte d’Ivoire. Firstly, we assessed genetic distances, phylogenetically informative sites found in more than one sequence contributing to branch ordering, *N*-glycosylation patterns, recombination events, selective pressures on West African BAGV sequences and their evolutionary phylogenetic relationships with previously available BAGV genomes. Secondly, to assess evidence of future BAGV codon adaptation to human house-keeping genes, a bio-informatics approach was used for analysis of codon adaptation biases.

## 2. Materials and Methods

### 2.1. Primers Design

RT-PCR amplification and complete genome sequencing were achieved by using overlapping primers designed by aligning available BAGV sequences from GenBank with Muscle algorithm implemented in the Mega 7.0 software (https://www.megasoftware.net/) [14]. All primer sets were designed using Primer3web^®^ software (version 4.0.0, Whitehead Institute for Biomedical Research, Cambridge, MA, USA) and submitted to a BLAST analysis on NCBI to avoid non-specific cross-reactions. Primers were synthetized by TIBMol-Biol (Berlin, Germany) and details are summarized in Table 1.

### 2.2. Virus stock preparation and RNA extraction

All 11 virus strains analyzed in this study were derived from the WHO Collaborating Center (*http://apps.who.int/whocc/Detail.aspx?cc_ref=SEN-5&cc_code=sen*) for arboviruses and viral hemorrhagic viruses in Senegal at Institut Pasteur de Dakar (Dakar, Senegal) (Table 2). Viral stocks were prepared by inoculating viral strains into *Aedes albopictus* (C6/36) cell line in Leibovitz 15 (L-15) growth medium (GibcoBRL, Grand Island, NY, USA) supplemented with 5% fetal bovine serum (FBS) (GibcoBRL, Grand Island, NY, USA), 10% Tryptose Phosphate and antibiotics (Sigma, Gmbh, Germany). BAGV infection was confirmed after 4 days of propagation by immunofluorescence assay (IFA) using specific hyper-immune mouse ascitic fluid, as previously described [15]. Cultures supernatants were collected for virus RNA isolation. Extraction of viral RNA from supernatants was performed with the QIAamp viral RNA mini kit (Qiagen, Heiden, Germany) according to manufacturer’s instructions. Extracted RNA was frozen at −80 °C prior to downstream applications.

### 2.3. RT-PCR and Sequencing

Real-time RT-PCR (Reverse Transcriptase-Polymerase Chain Reaction) was performed using the Quantitect^®^Probe RT-PCR Kit (Qiagen, Heiden, Germany) in a final volume of 25 μL following previously established protocols and primers [16]. Reverse-transcription was performed using the AMV kit (Promega, Madison, WI, USA) following manufacturer’s instructions and cDNA were stored at −20 °C. The polymerase chain reaction with each primer set was carried out in a final volume of 50 μL using the GoTaq^®^ DNA polymerase kit (Promega, Madison, WI, USA) according to manufacturer’s instructions. Briefly, 5 μL (around 10 µg) of cDNA was added to 45 μL of a RT-PCR mix containing 25 mM MgCl2, 10 mM of dNTP, 5X reaction buffer, 5 U Gotaq polymerase, 16.5 µL of nuclease-free water and 40 pmols of each primer (Sense and Antisense). PCR was carried using the following conditions: an initial incubation at 95 °C for 5 min, followed by 40 cycles of 95 °C for 1 min, 1 min at melting temperature of primers, and 72 °C according to the length of PCR product and 72 °C during 10 min. Subsequently, 5 μL of each PCR product was analyzed by gel electrophoresis on 1% agarose gels stained with ethidium bromide to check the size of amplified fragments by comparison to a DNA molecular weight marker (HyperLadder™ 1 kb, Bioline, Taunton, MA, USA). The DNA bands from the PCR amplification were purified (QIAquick Gel Extraction Kit, Qiagen, Heiden, Germany) and sequenced from both ends for each positive sample (Beckmann Coulter, High Wycombe, UK). Sequencing of the 5′ and 3′ termini of the viral genome was performed using a 5′ RACE kit (Invitrogen, Carlsbad, CA, USA) and a 3′ RACE kit (Roche, Basel, Switzerland) following the manufacturer’s protocols. Additional sequences representing strains from Central African Republic, India, strain related to Spanish wild bird’s outbreak in 2010, the ITV and the Ntaya virus were obtained from GenBank, with the following accession numbers, respectively: AY632545, EU684972, HQ644143, KC734549 and NC_018705.

### 2.4. Sequence Properties Analysis

Full-length genome sequences BAGV isolates were obtained by assembling overlapping nucleotide sequences using the Unipro UGENE software (http://ugene.net/download.html) [17]. Multiple alignments of full-genome sequences were carried out by using Muscle algorithm (http://www.drive5.com/muscle/) [18] within Unipro UGENE software. Based on these alignments, we investigated the genetic properties of these different isolates circulating in West Africa, such as genome length and location of main conserved amino acid motifs previously described in mosquito-borne flaviviruses (MBFVs) with sometimes mutations which include no physicochemical properties changes [3]. Comparatively, conservation of these motifs was also assessed in Culex flavivirus (CxFV) and Aedes flavivirus (AeFV) (insect-specific flaviviruses; (ISFs)) and in Modoc virus (ModV) and Rio Bravo virus (RBV) (Vertebrate-specific flaviviruses, also known as no known vector flaviviruses (NKVFs)). We also searched for evidence of informative amino acid sites among BAGV sequences using the DIVEIN web server (https://indra.mullins.microbiol.washington.edu/DIVEIN/) [19]. The genetic divergence between previously available BAGV complete genomes and new characterized sequences was also assessed at the nucleotide and protein levels.

### 2.5. Prediction of N-Glycosylation Sites

Prediction of N-glycosylated sites on the genome of BAGV were performed by submitting complete polyproteins on online version of NetNGlyc 1.0 Server (http://www.cbs.dtu.dk/services/NetNGlyc/). N-linked glycosylation is a post-translational event whereby carbohydrates are added to Asparagines, which occur in the consensus sequence Asn-Xaa-Ser/Thr, where Xaa is any amino acid except proline. “Potential” scores of predicted *N*-glycosylated sites across the protein chain from N- to C-terminal were illustrated using the default threshold of 0.5 and the “jury agreement” indicates how many of the nine networks support the prediction [20].

### 2.6. Prediction of Conserved Structural RNA Domains in 5′ and 3′ UTRs

The RNAz method [21] implemented in the Vienna RNA Websuite (http://rna.tbi.univie.ac.at/) [22] was used to detect thermodynamically stable and evolutionarily conserved structural RNA domains on complete non-coding regions of the 11 West African BAGV isolates characterized in this study and the isolates from Spain and CAR, because complete non-coding sequences are not currently available for the isolate from India. The RNAz method use an algorithm which testing a large set of well-known conserved structural RNA domains and reports a “RNA classification probability” or *p*-value as a measure of thermodynamic stability. Structural RNA domains with *p* > 0.5 are classified as stable [21]. Furthermore, the optimal secondary structures were predicted with a minimum free energy using the RNAalifold method [23] implemented also in the Vienna RNA Websuite that use a dynamic programming algorithm with RNA parameters as previously described [24]. Furthermore, previously described organization of conserved sequences (CS) [3] was analyzed on predicted secondary structures of the 3′ UTR, considering possible repetitions of these CS. Thus, a conserved sequence was considered as imperfect when it presented three or more differences with corresponding consensus sequence previously described [3], marked by a deletion, an insertion, or a substitution.

### 2.7. Phylogenetic Tree Inference

A Bayesian phylogenetic analysis for estimation of data quality and selection of the best-fit nucleotide substitution model were performed using Mega 6.0 (https://www.megasoftware.net/) with a discrete Gamma distribution (+G) with 5 rate categories. Thus, a total of 24 different nucleotide substitution models were tested and model with the lowest BIC score (Bayesian Information Criterion) was considered to describe the best substitution pattern. Further parameters as AICc value (Akaike Information Criterion, corrected) and Maximum Likelihood value (lnL) are also estimated [25]. A maximum likelihood tree was then constructed with complete polyprotein sequences from insect-specific flaviviruses, no known vector flaviviruses, tick-born flaviviruses, mosquito-borne flaviviruses, the 11 ORFs from new characterized West African BAGV isolates and BAGV sequences previously available from Spain (HQ644143-4, KR108244-6), India (EU684972) and CAR (AY632545). Tree was inferred using FastTree v2.1.7 (http://www.microbesonline.org/fasttree/) [26], where nucleotide substitution was modeled using General Time-Reversible with a proportion of invariant sites (GTR+I). Nodes were labeled with local support values, which were computed with the Shimodaira-Hasegawa test (SH-like) for 5000 replications. Topology was visualized by FigTree v.1.4.2 (http://tree.bio.ed.ac.uk/software/figtree/).

### 2.8. Recombination Detection

To prevent potential biases during phylogenetic inference due to recombination, all polyprotein sequences were analyzed using seven methods (RDP, GENECONV, MaxChi, BootScan, Chimaera, SiScan, and 3Seq) implemented in the Recombination Detection Program (RDP4beta 4.8) to uncover evidence for recombination events [27]. The disentangle recombination signals option was “on” and the linear sequence setting was used. The remaining settings were kept at their default values. Only events with *p*-values < 1 × 10^−6^ that were detected by four or more methods were considered to represent strong evidence for recombination using 100 permutations and the Bonferroni correction [28] implemented in the RDP4 program to prevent false positive results. A chi-square test was used to determine if the sequence identity between a recombinant isolate and a given parent was significantly different both inside and outside the recombinant region. In addition, a BootScan analysis including the recombinant and the parental strains determined above was also performed to confirm these putative recombination events. The occurrence of recombination in BAGV genomes was also investigated with a method called Genetic Algorithms for Recombination Detection (GARD) implemented in Datamonkey web server (http://datamonkey.org) [29], that estimates breakpoints based on a genetic algorithm. The statistical significance of putative breakpoints was evaluated through Kishino-Hasegawa (HK) tests; breakpoints were considered significant if their *p* value was <0.05. Separate Neighbor-Joining (NJ) trees were constructed for identified putative recombinant region and non-recombinant alignment partitions dictated by the breakpoint locations. Phylogenetic trees were inferred using the percentage of 1000 bootstrap replications under the appropriate model of nucleotide substitution.

### 2.9. Evaluation of Selection Patterns on ORFs

Recombination can mislead inference of positive natural selection if it is not properly accounted for. If recombination was identified, these potential recombinant sequences were excluded from further analyses to avoid inferential biases [30]. The non-synonymous/synonymous rate ratio (dN/dS) is a widely used method to detect positive selection. The statistical test dN/dS permitted to distinguish diversifying or positive selection (dN/dS > 1) from negative or purifying selection (dN/dS < 1). Positive selection is inferred when the rate of non-synonymous (dN) substitutions is higher than that of synonymous (dS) substitutions (dN > dS). Episodes of positive selection in each gene of BAGV were analyzed using methods of estimation among individual sites and internal sites on branches of the phylogenetic tree. For this, a total of 9 alignment partitions were performed corresponding to C, prM, E, NS1, NS2A, NS2B, NS3, NS4A, NS4B and NS5 proteins. As site model, we used the single-likelihood ancestor counting (SLAC) that estimated the difference between non-synonymous (dN) and synonymous (dS) rates per codon site at 0.1 significance level. The fast unconstrained Bayesian approximation (FUBAR) method which evaluated episodic positive selection at each site in the alignment at posterior probability ≥0.9 was also used [31]. The mixed effects model of evolution (MEME) was also conducted at a 0.1 significance level for estimation selective pressure changes among codon sites. Finally, branch-site random effects likelihood (Branch-site REL) analysis was used to evaluate evidence of diversifying selection on specific branches in the phylogenetic tree at a proportion of sites, considering *p*-values less than 0.05 as significant. All four methods were conducted with HyPhy package implemented in Datamonkey web server [29]. An episode of positive diversifying selection in concern of a region was considered if it was detected by at least two different methods.

### 2.10. Bayesian Analysis

The evolutionary analysis was performed using a strict clock GMRF Bayesian Skyride coalescent tree prior [32,33]. The GTR substitution model was used with 4 gamma rate categories. The Bayesian Markov Chain Monte Carlo (MCMC) algorithms using BEAST v1.8.4 (http://beast.community/) [34] were employed to estimate the rate of BAGV evolution from first isolation to 2014. MCMC analyses were run for 100 million generations, sampling every 100 thousand to ensure convergence of estimates. Population size (ESS) above 200 was assessed using the analysis program Tracer v1.6 (http://beast.bio.ed.ac.uk/Tracer). The posterior distribution of trees obtained from the BEAST analysis was also used to obtain the Bayesian maximum clade credibility (MCC) tree for these sequences generated by TreeAnnotator v.2.3.2 (http://beast.community/treeannotator) (from 100 million) after removing 10% of the runs burn-in and visualized by FigTree v.1.4.2.

### 2.11. Codon Adaptation Indexes to Human House-Keeping Genes

The Codon Adaptation Index (CAI) is a measure of the synonymous codon usage bias making comparisons of codon usage preferences in different organisms and assessing the adaptation of viral genes to their hosts [35,36]. CAI was applied in many recent studies involving humans and RNA viruses [37,38,39]. To know if there is evidence of BAGV adaptation for codon usage in humans, the CAI was calculated for each isolate. To calculate normalized CAI, full-length polyprotein sequences of West African BAGV isolates and previously available BAGV sequences from Spain were compared to that of human using CAIcal v1.4 program (http://genomes.urv.es/CAIcal/) [40]. First, we obtained a “raw” CAI (rCAI) and then, the CAI was normalized by the “expected neutral CAI” (eCAI) value based on 1000 random viral sequences using similar length, codon composition, GC-content and human amino acid usage. Indeed, a table for human codon usage containing the entirety of human coding genes is publicly available [41]. Based on this table, we created a new table where only the 3804 identified human housekeeping genes were considered [42]. Normalized CAI threshold was obtained by calculating rCAI/eCAI values and a value above ‘1’ is higher than neutral and considered as evidence of codon adaptation to the reference set of codon preferences [40]. CAI values obtained for BAGV were then compared to those of others MBFVs well known to infect humans such as Dengue virus (DENV), Usutu virus (USUV), WNV, Zika virus (ZIKV) and Yellow fever virus (YFV), NKV flaviviruses (ModV and RBV) and ISFs (CxFV and AeFV), using the non-parametric Wilcoxon test with R program. A *p*-value less than 0.05 was considered as significant. Sequences of tobacco mosaic virus (TMV) were compared to human codons and used as negative control to provide an example that results for codon adaptation to human house-keeping genes are robust and not false positives or anomalies. As there are no known cases of human infection, or evidence of human adaptation for TMV, we expected all sequences to have a lower CAI threshold than the calculated CAI.

## 3. Results

### 3.1. Genetic Diversity

In this study, a total of 11 full-genome sequences (10,954 bp) of West African BAGV isolates were obtained by sequencing overlapping PCR amplifications covering the genome and by using RACE (Rapid amplification of cDNA ends) techniques for the terminal ends and deposited in GenBank (www.ncbi.nlm.nih.gov/genbank/) (Accession numbers: MF380424-34) (Table 1). Analysis of new characterized BAGV complete open reading frames (ORFs) was performed at nucleotide and amino acid levels including previously available sequences from CAR (isolate DakArB209_CAR_1966, accession No. AY632545) and Spain (isolate Spain_H_2010, accession No. HQ644143) into multiple sequence alignments. The polyprotein length of the newly sequenced West African BAGV isolates was determined with respect to gene sizes (Table 3). Although the 5′ UTR was similar in length, the 3′ UTR of these West African isolates was either 10 nt or 137 nt longer than those of sequences from CAR and Spain, respectively.

In the 5′ UTR, positions 52, 55 and 93 had nucleotide changes that were distinguishable for West African isolates. Nucleotide changes A to C at position 52 and T to C at position 55 were seen in West African sequences, and a T to C change at position 93 was observed only in sequences of the isolates ARD54139_Dakar-Bango_SEN_1989 and ARA23139_Dezidougou_CI_1988. Interestingly, the 3′ UTR can be divided into three sections; a proximal highly variable section constituted by the 139 first nucleotides following the stop codon, a second highly conservative section located between nucleotide positions 140 and 434 and a moderately variable distal region comprising the last 142 nucleotides. In this distal section, 3′ UTR sequences of West African isolates presented insertions of 74 nt and 1 nt, compared to the isolates from CAR and Spanish (KR108244-6), respectively.

**Figure 1:**
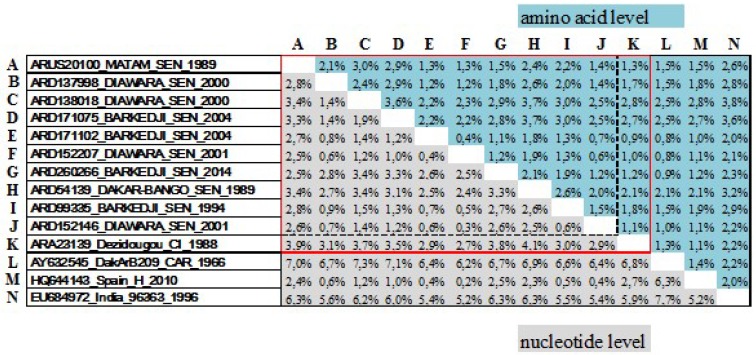
Genetic distances (in percentage) at nucleotide (gray) and amino acid (blue) levels between polyprotein sequences of Bagaza virus isolates characterized in this study (red line) and in comparison with previously available Bagaza virus sequences (Black line). Genetic distances between Senegalese isolates are encountered with zebra black line. Isolates name is labeled in the following format: Identification number, Origin, country code (SEN: Senegal, CI: Côte d’Ivoire, CAR: Central African Republic), and year of isolation; Except for Spanish and Indian Isolates (accession number, country, identification, and year of isolation).

Pairwise genetic distances of coding sequences were evaluated at nucleotide and amino acid levels between isolates characterized in this study and in comparison with previously available BAGV sequences (Figure 1). Nucleotide sequences of BAGV isolated from Senegal showed a mean distance of 1.9% ± 0.8% (0.3–3.4%). This lowest genetic distance was also apparent at amino acid level with a mean distance of 1.9% ± 0.6% (0.4–3.7%).

In comparison to sequence of the isolate ARA23139_Dezidougou_CI_1988 from Côte d’Ivoire, Senegalese BAGV isolates showed a higher mean distance of 3.4% ± 0.5% (2.7–4.1%) at nucleotide level. However, this highest genetic distance was less apparent at amino acid level with a mean distance of 1.7% ± 0.7% (2.7–4.1%). Furthermore, mean distances of 6.7% (6.2–7.3%), 1.3% (0.2–2.7%), 5.8% (5.2–6.3%) were recorded at nucleotide level between Senegalese BAGV isolates and the isolate from CAR, Spain, and EU684972__96363_India_1996, respectively while respective mean distances were 1.5% (0.8–2.5%), 1.7% (1.0–2.8%), 2.7% (2.0–3.8%) at amino acid level. A differentiation coefficient value of 0.17 was also observed between these West African BAGV isolates and previously available sequences.

### 3.2. Genetic Motifs and Informative Sites on BAGV Genome

Here, we described location of main conserved amino acid motifs on BAGV proteins using in silico analysis of complete genome sequences of the 11 West African BAGV isolates characterized in this study and sequences from India, CAR and Spain. Most of highly conserved amino acid motifs localized across E, NS1, NS3 and NS5 proteins of MVFs were identified in the BAGV genomes, sometimes with presence of conservative amino acid mutations (positions highlighted in Black) or non-conservative amino acid mutations (positions highlighted in red) (Table 4). Non-conservative amino acid motifs were observed between positions 667–675 in the envelope protein (E), 712–719 and 1120–1127 in NS1, 1722–1728 and 1759–1766 in NS3 and between positions 2734–2741 in NS5. A Leucine (L) insertion was observed at position 674 (E) of the polyprotein of all BAGV isolates.

In protein NS1, all analyzed motifs were conserved, but nc T713P and T713A were observed in sequences of isolates ARD171102_Barkedji_SEN_2004 and EU684972_96363_India_1996, respectively. The BAGV isolate ARD54139_Dakar-Bango_SEN_1989 also contains nc P1127T. In NS3, the conserved motif identified at positions 1722–1728 contains nc A1723P and P1724L for BAGV isolates ARD137998_Diawara_SEN_2000 and ARD138018_Diawara_ SEN_2000, whereas nc L1722S is present in ARD138018_Diawara_SEN_2000, and ARD171075_Barkedji_SEN_2004. A non-conserved motif at positions 1759–1766 contained nc F1766L in all BAGV sequences analyzed and isolate ARD138018_Diawara_SEN_2000 contained additional nc T1765P and D1785Y. A non-conserved motif in NS5 at positions 2734–2741 contained nc T2738N in all BAGV sequences. BAGV isolate ARD54139_Dakar-Bango_SEN_1989 has two supplementary mutations S2734W and S2737P. In addition, these MBFVs amino acid motifs were also mostly conserved in BAGV, NKVFs and CxFV (ISFs) than in AeFV (ISFs).

**Figure 2:**
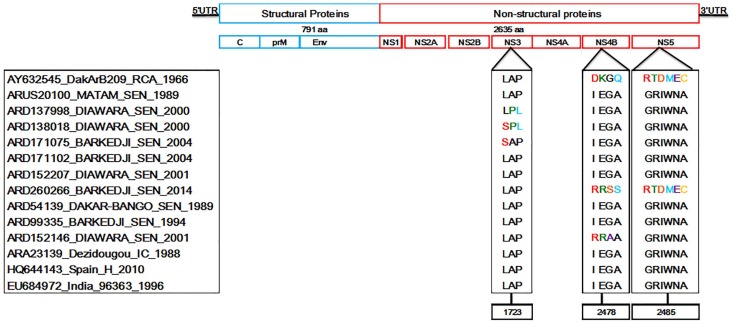
Determination of informative sites on polyprotein sequences of Bagaza virus isolates. Amino acid positions with non-conservative mutations are highlighted in different colors. Location of each motif is identified by corresponding positions on Bagaza virus genome.

Non-conservative amino acid mutations on the BAGV polyprotein might be associated to phenotypic differences of BAGV isolates. In addition, the presence of phylogenetically informative sites was assessed on the DIVEIN web server. The identified site LAP is harbored by the conserved motif LAPTRVV previously identified in NS3 protein of flaviviruses [3] and presents nc mutations in the genome of three BAGV isolates. In addition, phylogenetically informative sites IEGA and GRIWNA identified in NS4B and NS5, showed combined variations in the genome of the CAR isolate (DKGQ and RTDMEC, respectively) and the Senegalese BAGV isolates ARD152146_Diawara_SEN_2001 (RRAA and GRIWNA, respectively) and ARD260266_Barkedji_SEN_2014 (RRSS and RTDMEC, respectively) (Figure 2).

### 3.3. Predicted N-Glycosylated Amino Acid Sites

**Figure 3:**
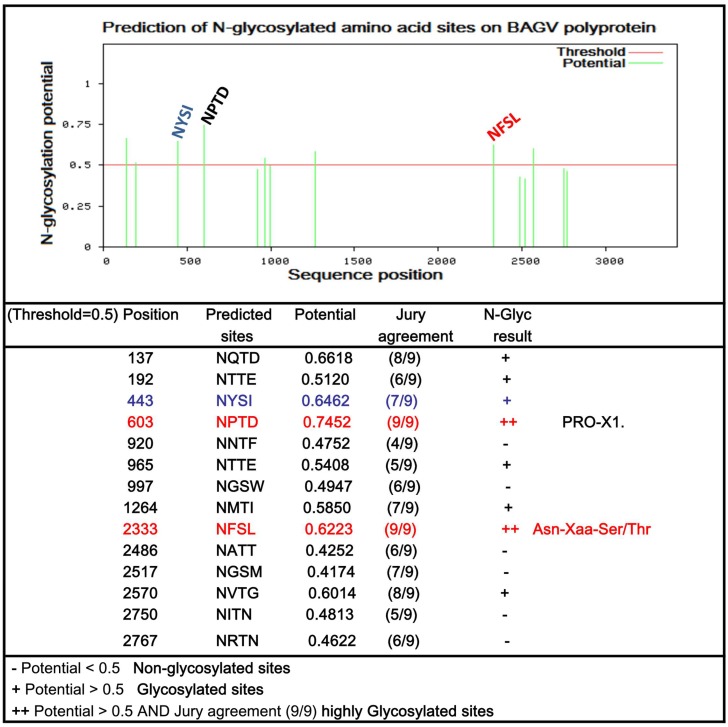
Prediction of *N*-glycosylation on Bagaza virus genome. Predictions were performed using the NetNGlyc 1.0 server. A position with a potential (green vertical lines) crossing the threshold (horizontal red line at 0.5) is predicted glycosylated. The “potential” score is the averaged output of nine neural networks and the “jury agreement” indicates how many of the nine networks support the prediction. The N-Glyc Result column shows one of the following outputs for predictions. N-glycosylated sites highly predicted by the nine networks (potential > 0.5 and jury agreement of 9/9) are highlighted in red and the site previously reported as virulence factor on E protein of flaviviruses is colored in blue.

Prediction of *N*-glycosylation sites was performed using complete genome sequences of the 11 West African BAGV isolates characterized in this study and sequences from India, CAR and Spain on the DIVEIN web server. The “potential” score represents the averaged output of nine neural networks and the “jury agreement” indicates how many of the nine networks support the prediction. In total, eight *N*-glycosylated motifs were identified in the BAGV genome (potential > 0.5) including two highly probable sites (potential > 0.5 and jury agreement of 9/9). Despite high potential (0.7452) and jury agreement (9/9), the motif (Asn-X-Thr) NPTD identified at position 603 was not considered to be glycosylated because it contained a Proline known to preclude the *N*-glycosylation by rendering inaccessible the Asparagine in the majority of cases (Figure 3). This motif was in the domain III region of the E protein of all BAGV isolates. However, a second (Asn-X-Ser) motif NFSL was highly predicted (score 0.6223 (9/9)) and suggested an N-linked glycosylation site at the residue Asn-2333 in the NS4B protein. Interestingly, we also found six others probable N-glycosylation at different positions on the BAGV polyprotein including one site (NYSI) harboring, the NYS motif at the 443th position (153th position of the E protein), previously described as a virulence factor for WNV and DENV.

### 3.4. Predicted Structural RNA Domains on UTRs of BAGV Genome

Assessment of thermodynamically stable and evolutionarily conserved structural RNA domains was performed using complete non-coding sequences of the 11 West African BAGV isolates characterized in this study and the isolate from Spain. The RNAz method implemented in the Vienna RNA Websuite was used to identify conserved structural RNA domains in the UTRs of BAGV characterized by a *p* > 0.5. Using the RNAz method, highly conserved structural RNA domains was not identified in the 5′ UTR of BAGV genome while a total of four highly conserved structural RNA domains were determined in the 3′ region with respective classification probabilities of 0.671490, 0.994641, 0.976295 and 0.846482 (Figure S1).

**Figure 4:**
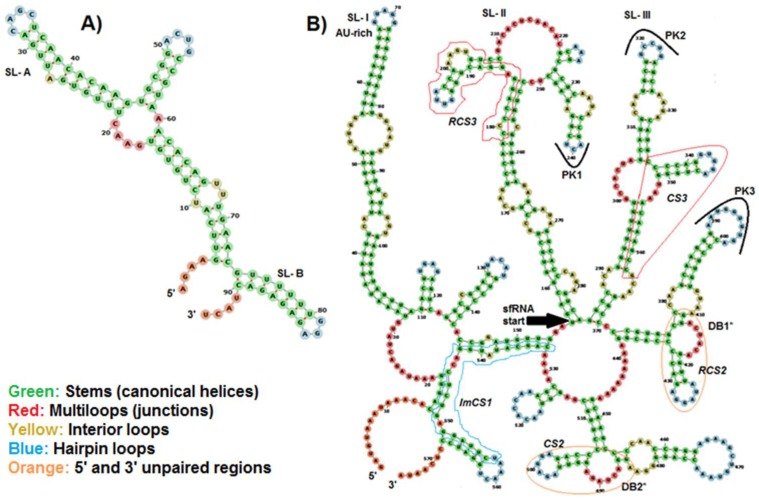
Secondary structure predicted from 5′ UTR (**A**) and 3′ UTR (**B**) sequences of Bagaza virus. Prediction was performed using the RNAalifold method implemented in the Vienna RNA Websuite. Sub-genomic RNA structure (sfRNA) of the 3′ UTR is organized as follows: RCS3-CS3-RCS2-CS2-ImCS1. Stem-loops (SL), dumbbell structures (DB*), pseudoknots (PK) and short conserved loops (CS), repeated conserved loops (RCS) are identified in the figure.

However, the RNAalifold method implemented in the Vienna RNA Websuite server predicted that, as in the genome of other members of the genus flavivirus, BAGV has a shorter 5′ UTR (≈100 nt), consisting of a pair of conserved stem-loops (SL-A and SL-B) (Figure 4). SL-A serves as promoter of viral polymerase activity followed by a shorter loop which contains a cyclisation sequence upstream of the 5′ AUG (SL-B). The secondary structure of BAGV’s 3′ UTR could be divided in three parts; a highly variable domain 1 following the stop codon and consisting in an AU-rich stem-loop (SL-I), a second domain 2 with highly conserved sequence and two stem-loops (SL-II and SL-III) and dumbbell structures (DB1 and DB2), and the moderately conserved distal domain 3 which contains the complementary cyclisation elements. In the intermediate domain, the SL-II presented a pseudoknot PK1 preceding a short conserved loop (RCS3). This structural motif was repeated in a stem-loop SL-III with PK2 and CS3. These stem-loops were followed by dumbbell structures DB1 and DB2 that presented conserved loop RCS2 connected with a pseudoknot PK3 and its repetition CS2, respectively [43]. Thus, organization of conserved sequences on consensus secondary structure of BAGV’s 3′ UTR was structured RCS3-CS3-RCS2-CS2-ImCS1. Indeed, CS1 was imperfect (ImCS1) only on sequences of West African BAGV isolates with a total of nine substitutions compared to the corresponding consensus sequence previously described [3].

### 3.5. Maximum Likelihood Tree

**Figure 5:**
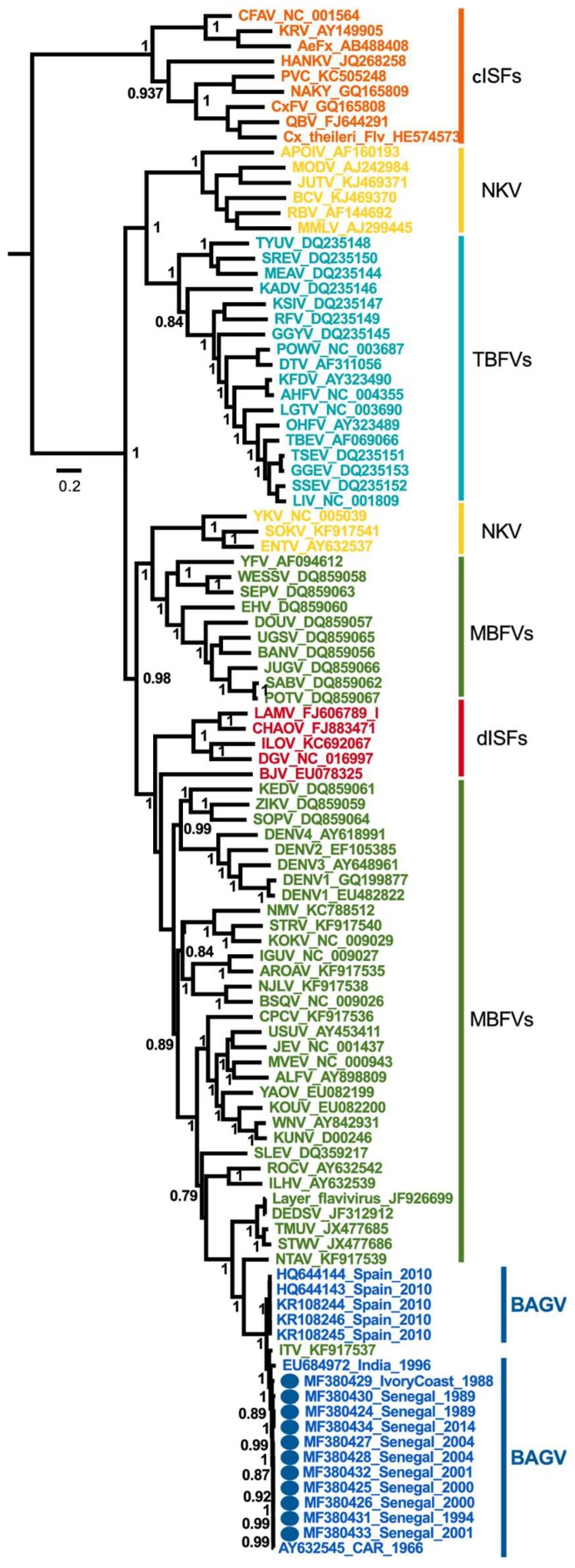
Maximum Likelihood (ML) tree based on complete polyprotein sequences of Bagaza virus isolates circulating in Senegal and Côte d’Ivoire from 1988 to 2014. The tree is midpoint-rooted, nodes are labeled with local support values computed using the Shimodaira-Hasegawa (SH) test for 5000 bootstrap replications, species names are color-coded as follows: new characterized BAGV isolates—dark blue with dots; previous sequences of BAGV—dark blue; mosquito-borne flaviviruses (MBFVs)—green; dual-host affiliated ISFs (dISFs)—red; no Known Vector (NKV) flaviviruses—yellow; tick-born flaviviruses (TBFVs)—light blue; classical ISFs (cISFs)—Orange.

The Bayesian phylogenetic analysis for estimation of data quality and selection of the best-fit nucleotide substitution model were performed using Mega 6.0 with a discrete Gamma distribution (+G) with 5 rate categories. The General Time-Reversible with a discrete Gamma distribution and a proportion of invariant sites (GTR+I) was the best nucleotide substitution model for our sequences data presenting score values of 69,924.128, 69,453.449 and −34,680.714 for BIC, AICc and lnL criteria, respectively. The maximum likelihood (ML) tree was inferred using FastTree v2.1.7 [26] on our total data set including the 11 complete polyprotein sequences of West African BAGV isolates circulating in Senegal and Côte d’Ivoire from 1988 to 2014, the 5 BAGV sequences from Spain, the BAGV sequences from India and CAR and complete polyproteins from different flaviviruses, with 10,281 bp alignment length (Figure 5). A GTR+I model was used, as selected by Bayesian criteria. Nodes were labeled with local support values computed with 5000 bootstrap replications using the Shimodaira-Hasegawa (SH) test. The phylogeny of complete BAGV genome sequences presented evidence of a single BAGV phylogenetic group. Furthermore, we observed also that Israel meningo-encephalitis turkey virus (ITV) was closed to BAGV in genetic relatedness [11].

### 3.6. Evidence of Recombination Events

**Figure 6:**
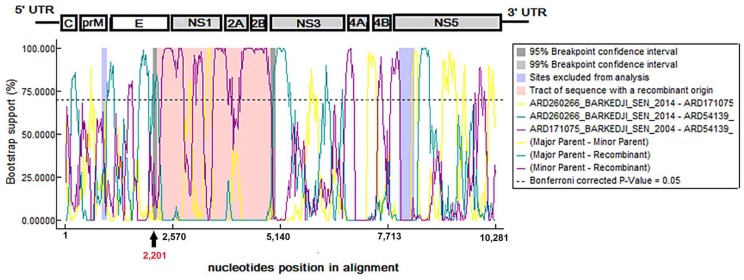
Recombination analyses of the full genome of Bagaza virus. Evidence of only one recombination event was observed and involved the Senegalese isolate ARD54139_DAKAR-BANGO_SEN_1989. The genomic region with a recombinant origin, ranging from the E protein to the NS2B, is colored in pink. The breakpoint identified at nucleotide position 2201 of E protein and supported by high bootstrap value and significant *p*-values (LHS: 0.024 and a RHS: 0.001) is colored in red.

Given the major implications of recombination events for evolution, pathogenicity, or diagnosis of non-segmented positive RNA viruses like flaviviruses [44], it is clearly important to determine their occurrence in the BAGV genome. The RDP4beta 4.8 program used for assessment of recombination events on complete polyprotein sequences [27] revealed evidence of only one highly credible recombination event from the E protein to NS2B, with estimated breakpoints at positions 2202 and 4908 of BAGV genome. This recombination event involved the isolate ARD54139_Dakar-Bango_SEN_1989 originating from Saint-Louis, in the North of Senegal (Figure 6). Considering the isolates ARD260266_Barkedji_SEN_2014 and ARD171075_Barkedji_SEN_2004 as respective minor and major parents of the isolate ARD54139_Dakar-Bango_SEN_1989 (Similarity of 98.8% and 97%, respectively), this recombination event was found by RDP, GENECONV, Bootscan, Maxchi, Chimaera, SiSscan and 3Seq methods and supported by significant *p*-values of 3.09 × 10^–16^, 9.23 × 10^−12^, 7.36 × 10^−13^, 8.45 × 10^−7^, 1.59 × 10^−7^, 3.60 × 10^−8^ and 1.17 × 10^−12^, respectively. The BootScan and GARD analyzes identified one significant recombination breakpoint at nucleotide position 2201 corresponding to the E protein, supported by a LHS *p*-value of 0.024 and a RHS *p*-value of 0.001.

**Figure 7:**
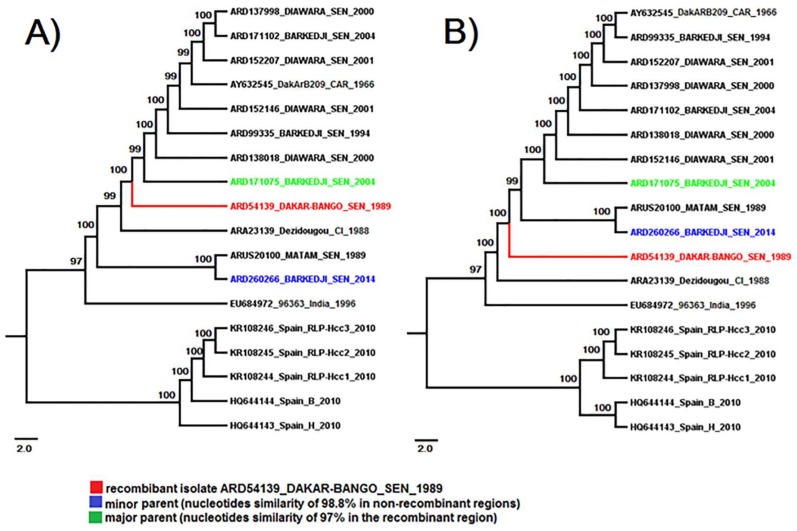
Neighbor-joining (NJ) phylogenetic trees inferred for recombinant genomic region (**A**) and non-recombinant fragments (**B**) significantly identified on full genome of Bagaza virus by the seven methods in RDP4 program. Trees were constructed using 1000 bootstrap replicates and midpoint rooted. The putative recombinant isolate is marked by the red color in the trees and the corresponding minor and major parental sequences are colored in blue and green, respectively.

This breakpoint divides the BAGV genome into two regions: one that encodes the structural proteins and another that encodes the non-structural proteins. Phylogenetic trees were constructed using 1000 bootstrap replications and midpoint rooted for clarity only (Figure 7). This recombination event led to a mismatch between NJ phylogenetic trees constructed using comparison of nucleotides sequences of recombinant (positions 2202–4908) and non-recombinant genomic regions (positions 1–2201 and 4909–10,281).

### 3.7. Positive Selection Pressures

The structural and non-structural coding regions were analyzed separately for estimation of sites and branches under positive diversifying selection, applying four different methods to ensure consistency of these events along of BAGV sequences. Using this approach, we found several sites under strong negative selection and most of them were in the E, NS3 and NS5 proteins (Table 5). However, the significant evidence (*p* < 0.1) of episodic positive selection was obtained for all the coding genes, except for the prM, NS2B and NS4A regions. All positively selected sites estimated by the FUBAR model (posterior probability ≥ 0.9) were also identified by the MEME method (*p* < 0.1). Thus, an important number of positively selected sites were detected; interestingly, the majority of such sites were in the E, NS1 and NS5 proteins. Branch-site analysis showed also a total of 11 branches evaluating under positive selection (*p* < 0.05) and the highest proportion was in the E and NS1 proteins.

### 3.8. Phylodynamics of Bagaza Virus

**Figure 8:**
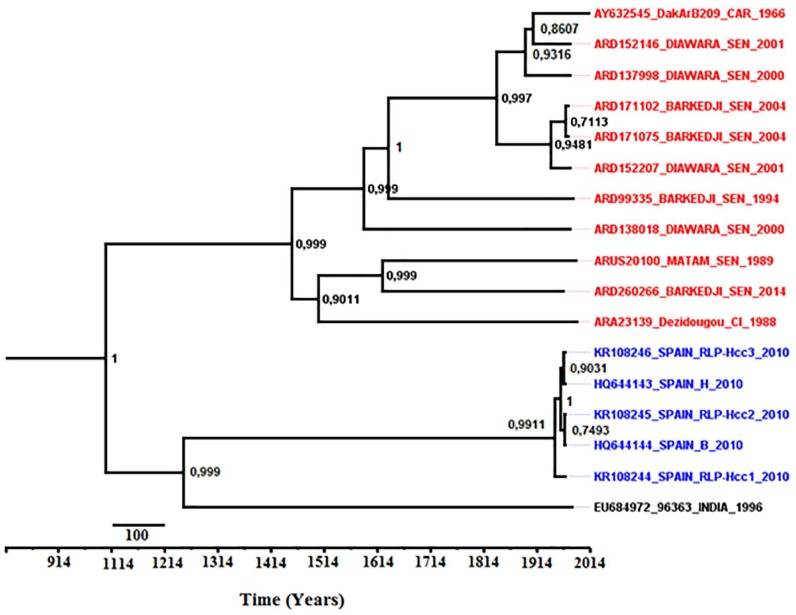
Maximum Clade Credibility (MCC) tree inferred for concatenated sequences from C, E, NS1, NS2A, NS3, NS4B and NS5 proteins of Bagaza virus isolates. Consensus MCC tree on which nodes were supported by posterior’s values and tree has been rooted with estimated times of emergence from the most recent common ancestor (TMRCA). Names of African BAGV isolates are highlighted in red, names of Spanish isolates in blue and name of the Indian isolate in black.

MCMC convergence was obtained for three independent runs with 100 million generations, which were sufficient to obtain a proper sample for the posterior at MCMC stationarity assessed by effective sample sizes (ESS) above 200 for each gene. Furthermore, the evolutionary rates (μ) and the highest posterior densities (HPD with 95% of confidence interval) were 1.226 × 10^−3^ (1.271 × 10^−3^–1.178 × 10^−3^), 4.218 × 10^−7^ (4.262 × 10^−3^–4.168 × 10^−3^), 3.181 × 10^−3^ (3.224 × 10^−3^–3.125 × 10^−3^), 1.092 × 10^−3^ (1.142 × 10^−3^–1.044 × 10^−3^), 3.910 × 10^−3^ (3.951 × 10^−3^–3.882 × 10^−3^), 1.921 × 10^−3^ (1.970 × 10^−3^–1.868 × 10^−3^) and 6.430 × 10^−3^ (6.480 × 10^−3^–6.386 × 10^−3^) substitutions/site/year for C, E, NS1, NS2A, NS3, NS4B and NS5, respectively. The Bayesian MCC analysis for proteins demonstrated one phylogenetic group of BAGV evolving from the most recent common ancestor (MRCA) originating in India during the 13th century (Figure 8). The West-African isolates diverged from the MRCA since the 15th century while the Spanish isolates had a recent divergence from the MRCA during the 20th century. In addition, the median root date estimates and 95% Bayesian credibility interval indicated that C, E, NS1, NS2A, NS3, NS4B and NS5 proteins diverged, respectively, 398 (93–3358), 1577 (267–18,784), 938 (16–9092), 308 (81–3043), 1626 (284–17,185), 946 (136–11,489) and 4525 (535–55,707) years ago from the MRCA.

### 3.9. Codon Adaptation Indexes of Viral Coding Genes

**Figure 9:**
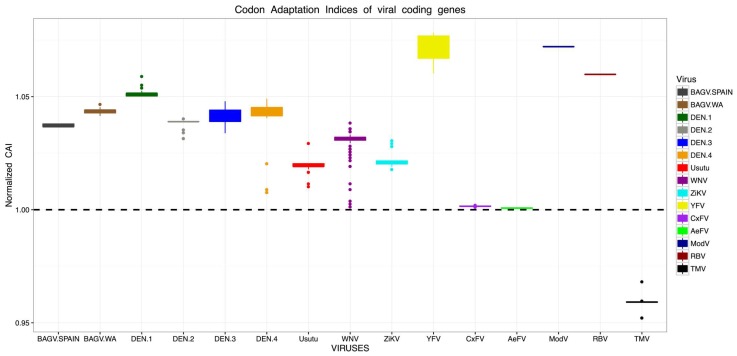
Evidence of Bagaza virus codon adaptation to human house-keeping genes. Normalized CAI values obtained with complete polyprotein sequences of Spanish (dark grey) and West African (brown) Bagaza virus isolates were compared to CAI of DENV (1 dark green, 2 grey, 3 bleu, 4 orange), USUV (red), WNV (dark purple), ZIKV (cyan), YFV (yellow), CxFV (purple), AeFV (green), Modv (dark blue), RBV (dark red) and TMV (black). CAI value > 1 (the black dashed line) was considered as evidence of codon adaptation to human house-keeping genes.

Evidence of BAGV adaptation to human house-keeping genes was analyzed by calculating CAI indices using complete coding polyprotein sequences of West African BAGV isolates and BAGV sequences available from Spain, in comparison to other MBFVs such as DENV, USUV, WNV, ZIKV and YFV, NKV flaviviruses (ModV and RBV) and ISFs (CxFV and AeFV). CAI values > 1 were obtained for polyprotein sequences of all BAGV isolates. Thus, there is evidence that BAGV could have adaptation to the human genes (Figure 9). ModV(mean CAI: 1.072 and median CAI: 1.072), RBV (mean CAI: 1.059 and median CAI: 1.059) and YFV (mean CAI: 1.075 and median CAI: 1.072) showed the highest CAI values for human housekeeping genes and were significantly different to Spanish and West African BAGV isolates (Wilcoxon Rank Sum Test, *p*-values ranging from 0.0001 to 1.028 × 10^−7^). Compared to those of Spanish isolates, sequences of West African BAGV isolates presented significantly higher CAI values (mean CAI: 1.044 and median CAI: 1.044, Wilcoxon Rank Sum Test, *p*-value < 0.002). In addition, they were also higher than DENV serotype 2 (DENV-2) (Wilcoxon Rank Sum Test, *p*-value < 1.4 × 10^−6^). However, CAI values of West African BAGV isolates were lower than those of DENV-1 (Wilcoxon Rank Sum Test, W = 231, *p*-value = 2.869 × 10^−6^) and comparable to CAI values given by DENV-3 and DENV-4 (Wilcoxon Rank Sum Test, W = 3258, *p*-value = 0.06477 and W = 824, *p*-value = 0.3463, respectively). Interestingly, CAI values of West African isolates were also significantly higher than those obtained for other MBFVs well known to infect human such as USUV (Wilcoxon Rank Sum Test, *p*-value < 6.796 × 10^−9^), WNV (Wilcoxon Rank Sum Test, *p*-value < 2.718 × 10^−10^), ZIKV (Wilcoxon Rank Sum Test, *p*-value < 1.67 × 10^−8^) and ISFs (means CAI: 1.0015 and 1.0006 and median CAI: 1.0015 and 1.0006 for CxFV and AeFV, respectively) which showed low evidence for codon adaptation towards human housekeeping genes (Wilcoxon Rank Sum Test, *p*-value < 2.328 × 10^−16^). Although CAI results for ISFs were significantly lower to human housekeeping genes, we did not find any significant difference between CxFV and AeFV codon adaptation. Compared to codon usage of human genes, sequences of tobacco mosaic virus (TMV) showed mean and median CAI values of 0.9587 and 0.9592, respectively.

## 4. Discussion

With an increasing number of emergent and re-emergent pathogens involved in human encephalitis, it is important to try to better understand which viruses have a potential to emerge causing human infection in the future. Since its first isolation, BAGV was only detected in mosquito pools collected in the field during entomological investigations in West and Central Africa and in India [12]. However, in 2010, BAGV was identified as the cause of an encephalitis outbreak in wild birds circulating in Southern Spain [9]. In a possible host-switching event [45], BAGV could acquire future adaptation to other vertebrates such as humans [46]. In this study, genetic properties of BAGV isolates circulating in West Africa, the evolutionary phylogeny of BAGV and evidence of BAGV adaptation to human house-keeping genes were evaluated in comparison with different flavivirus groups.

Genomes of 11 West African BAGV strains isolated from mosquito pools collected in the field from 1988 to 2014 showed similarities in terms of gene lengths when compared with polyprotein sequences of previously available isolates from CAR and Spain. 

Low amino acid distances observed between West African isolates (<2%) in comparison with previously non-West African sequences (<3%) combined with the weak coefficient of differentiation (<0.2) revealed evidence of a low genetic diversity of BAGV sequences analyzed in this study as previously described [9]. In addition, the West African BAGV isolates were more closely related to the CAR isolate. Genome sequences originating from BAGV isolates from other geographic locations would be helpful to understand if this low diversity is secluded to West-Africa.

Although the 5′ UTR was conserved between isolates, the 3′ UTR of West African isolates varied in terms of length and structure. As in other mosquito-borne flavivirus genomes, BAGV genome harbored structural RNA domains both in 5′ and 3′ UTRs which play a major role in flaviviral replication and interactions with host proteins and regulate cellular response to infection [47,48]. However, differences in determination of structural RNA domains in 5′ UTR between the RNAz and the RNAalifold methods used in this study could be attributed to variations in algorithm of analysis used by each method [21,22,23,24]. The small subgenomic RNA (sfRNA) identified in the 3′ UTR of BAGV is generated through incomplete degradation of the viral genome by cellular 5′-3′ exonuclease XRN1 [49,50] and plays an important role in viral pathogenicity [49] and modulation of host responses [51,52].

In addition, the stable 3’ terminus region of the sfRNA following the dumbbell structures (DB1 and DB2) and complementary to the 5′ terminus of the 5′ UTR, was shown to be necessary in genomic RNA cyclisation for viral replication and translation [46]. The sfRNA can be in competition with the 3′ UTR of genomic RNA in binding to proteins of viral replication complexes (RC) [53] and/or cellular machinery [54]. Thus, it slows down the replication or translation and assembly of particles [51].

The 3′ UTR region is important for translation and replication of the RNA genome through interactions with viral and host proteins, genome stabilization, and RNA packaging [55]. A better understanding of the potential impact of 3′ UTR variations in replication of BAGV could be important in the study of mechanisms implicated in their pathogenicity [56,57]. 

Most motifs linked to virulence previously described in these proteins of other MBFVs were conserved among BAGV isolates. However, some non-conservative mutations were identified in E, NS1, NS3 and NS5. In general, non-conservative amino acid mutations (nc) are spontaneous, rare, and hazardous, and then represent the main causes of genetic diversity. Thus, non-conservative mutations observed on BAGV genome could modulate viral phenotypes of particular isolates in mechanisms such as virus cell entry, replication, production of viral particles, and assembly, and cause modifications in post-translational regulation as previously demonstrated for other flaviviruses such as DENV [58,59,60]. The E protein is involved in cell receptor recognition, attachment, cell fusion, tropism, and virulence [58]. NS1 is the most conserved non-structural protein of flaviviruses. Associated with the other non-structural proteins, the NS1 protein plays an important function in viral replication and assembly and viral escape to host innate immune response [61]. The NS3 protein is the main component of the replication machinery and ensures multiple functions in viral evasion to host antiviral response and in production and assembly of infectious viral particles [62]. The NS5 protein is the largest viral protein that serves as the RNA-dependent RNA polymerase (RdRp) and performs multiple functions essential for viral replication, including processing the viral polyprotein, replicating the viral RNA. Sharing these motifs of virulence mostly with MBFVs, NKVFs and CxFV than with AeFV showed that BAGV could be more closely related to MBFVs transmitted by Culex mosquitoes and could explain frequent BAGV isolations mainly from mosquitoes of Culex genus and its capacity to infect vertebrates such as wild birds [1,4,5,9,10]. In addition, The West African BAGV isolates characterized in our study were mainly isolated from *Culex poicilipes* and *Culex neavei* mosquitoes which have been reported as potential vectors for flaviviruses such as WNV [63]. *Culex neavei* was also found as a competent vector able to transmit flaviviruses such as USUV and WNV [64,65]. Despite no available data on *Culex poicilipes* competency to transmit flaviviruses, these two mosquito species belonging to Culex genus could play an important role in natural transmission of BAGV to vertebrates such as wild birds since another member of the Culex genus, *Culex tritaeniorhynchus*, has been found competent to transmit BAGV to mice [8].

The phylogenetically informative sites identified on the BAGV genome located mainly in NS3, NS4B and NS5 proteins, respectively, could have a considerable impact in viral fitness on host for corresponding West African isolates. In addition, the prediction of the *N*-glycosylated sites at different positions on BAGV genome such as Asn2333 in NS4B and the NYSI motif at 153th position of the E protein showed that post-translational modifications may influence acquisition or loss of capacity in mechanisms such as pathogenicity, evasion of innate immune pathways. Indeed, flaviviruses NS4B plays an important role in replication of viral RNA facilitating the formation of replication complexes and modulating host innate immune response such as interferons, microRNAs and RNA interference, formation of stress granules and the unfolded protein responses [66,67,68]. A previous study had shown that *N*-glycosylation of NS4B of DENV does not affect the protein stability but causes a considerable reduction in efficiency of viral production [69]. Presence of a glycosylation site and an informative site in the viral NS4B protein could influence the efficiency of viral replication and the outer shape of the virion. The presence of the N-linked glycosylation motif NYS had been previously reported at 67/153th and 154th on the E protein of DENV and WNV (lineage 1 strains and some neuroinvasive lineage 2 strains), respectively, involving in receptor binding, viral morphogenesis, viral infectivity, and tropism [70,71,72,73,74]. Since glycosylation is a means of evasion to immune recognition within the host by masking particular antigenic sites from recognition by neutralizing antibodies, it could increase the diversity of the glycosylation on viral proteins [75,76]. Nevertheless, it could be important in future studies to determine whether the predicted glycosylation sites are really used (asparagine-linked) using specific enzymatic digestion by Endo H and peptide *N*-glycosidase (PNGase F) [77]. Our data suggest the ability of BAGV to develop phenotypically important variations and potentially adaptation to new vertebrate hosts such as humans. However, to understand better the impact of variation on these predicted *N*-glycosylation sites and the identified phylogenetically important variations would require in vitro studies with reverse genetically engineered infectious clones on mosquito or mammalian cell lines and in vivo experiments in mosquitoes or in animal models like mouse [78,79]. However, antibodies against BAGV proteins or infectious clone are currently not available for BAGV.

The identification of natural recombination events between virus isolates is important for our understanding of virus evolution. In our study, we identified a recombination event in the E protein BAGV. Recombination was documented in other members of the mosquito-borne flavivirus group [80,81], but had not yet been demonstrated to occur in BAGV. Identification of recombination breakpoints and the graphical detection of conflicting phylogenetic signals gave confirmation of this recombination event in E protein of the Senegalese isolate ARD54139_Dakar-Bango_SEN_1989 as previously described for ZIKV [74,82]. Nevertheless, the precise molecular mechanisms of the template switches are unknown. The E protein is highly important because it encodes the most important antigen with regards to virus biology and humoral immunity. Therefore, large-scale genetic changes in this region, as might be brought about by recombination, could have significant impact on virus phenotype [44].

The estimation of the selection pressures acting on each protein of BAGV demonstrated episodes of strong negative selection in functionally important proteins. These results suggested frequent purging of deleterious polymorphisms in the BAGV genome that could be associated with accumulation of synonymous mutations during BAGV transmission [83]. However, location of more significant episodes of positive selection in the E, NS1 and NS5 proteins indicated that they could represent preferential selection targets during BAGV evolution [84]. Indeed, the E protein of flaviviruses plays a crucial role in early steps of host cell binding and viral entry and represents a main target for immune responses influencing antigenic response and positive selection on the E protein is a hallmark of the emergence of flaviviruses [85,86]. Positive selection episodes have been also previously reported for the DENV-3 capsid, however, the impact needs to be further investigated [87]. Likewise, non-structural proteins could also be targets of positive selection.

The NS1 protein is essential for viral RNA replication, is involved in immune system evasion, and represents the major positive selection target during speciation of arthropod-born flaviviruses such as DENV and ZIKV [88]. NS2A and NS4B proteins have been shown to antagonize the interferon response during DENV infection [89] and changes in these regions would be evolutionary advantageous selecting for BAGV strains with strong innate immunity suppression mechanisms. Mutations in the NS4B protein were also seen to modulate several phenotype mechanisms of flaviviruses, such as pathogenesis [90], viral adaptation [91], replication [68], neurovirulence [66] and host preferences [92]. Thus, presence of positively selected sites in NS4B of BAGV isolates could have major impact in its natural evolution.

NS3 and NS5 proteins are crucial for viral replication, since non-conservative changes in these regions could modify process of protease and ATPase/helicase functions of NS3 protein [93] and RNA polymerase activity of NS5 protein [94]. These several polymorphic amino acid coding sites in the BAGV genome suggest that these proteins may be experiencing relatively adaptive changes in the natural evolution and they should be prioritized in future experimental studies.

Despite the evidence of a single phylogenetic group for BAGV sequences analyzed in our study, the evolutionary rates are expected in accordance to proteins functions; the NS5 representing the polymerase and the most conserved protein [86]. The inferred Bayesian MCC trees indicated a single introduction of BAGV into Europe and Africa from India, contrary to other African flaviviruses as WNV [95] and ZIKV [74], suggesting an Indian origin of BAGV. Estimated times from the MRCA suggested a distant origin of West African BAGV sequences analyzed in this study from the 15th century. Thus, further phylodynamic analyzes based on more complete sequences could be interesting for determining geographical pathways and potential evolution patterns in correlation with BAGV spread from India to African and European continents.

The Codon Adaptation Index (CAI) represents a reliable bio-informatics approach to measure the synonymous codon usage bias and to assess the adaptation of viral genes to their hosts [96]. Flaviviruses can infect and replicate in hosts of different phyla. Therefore, their versatility in gene expression and protein synthesis and changes in the viral RNA genome could affect the fitness of the virus in a specific host relating to dinucleotide frequencies, codon preferences, and codon pair biases [97,98,99]. Nevertheless, ecology, different virus-host relationships, biogeographical migrations of flavivirus species and genetic differences may explain observed differences in flaviviral codon usage preference to human housekeeping genes [98,100,101]. In particular, NKV flaviviruses were only isolated from vertebrates and are maintained in nature by horizontal transmission between vertebrate hosts [102,103]. Although ISFs were thought to sustain their populations in their respective insect vectors in the absence of mammal reservoirs, so lower translational efficiency in vertebrates could be expected [97,104].

In addition, the highest CAIs of YFV and DENV could be related to their long histories of infection in humans [105]. Indeed, YFV and DENV are maintained in endemic and sylvatic cycles, which conducted to repeated epidemics for more than one hundred years. However, YFV showed generally a higher virulence in human infections, particularly when it is compared to DENV infections reported in Africa [106]. This could explain the higher CAI values of YFV towards codon usage of the human housekeeping genes.

With evidence of adaptation to human house-keeping genes, BAGV could be potential cause of infection in vertebrates, such as humans. Considering the highest CAI values of West African BAGV isolates when compared to isolates responsible of the Spanish wild bird’s outbreak in 2010 [9], BAGV adaptation to vertebrate species such as birds could have led to an extension of adaptation to other species as shown in a previous virus study [46]. Interestingly, West African BAGV isolates showed a higher evidence of codon adaptation than MBFVs well known to infect humans, such as WNV which is a major cause of human encephalitis in USA and responsible of recent outbreaks in Europe [107] and ZIKV associated with microcephaly in fetuses and newborns during the outbreak in Brazil in 2015 [91]. Thus, further comparison of codon adaptation indexes of other BAGV genomic regions, such as the 3′ UTR, among isolates that differ in biological, ecological, and genetic characteristics could help to characterize the evolutionary adaptation of BAGV genomes to vertebrate hosts [46,108].

Nevertheless, to ensure the potential of BAGV to be involved in human encephalitis cases, it would be important to evaluate its pathogenicity on human induced pluripotent stem cell lines (iPSC) capable of differentiating into brain microvascular endothelial cells (BMECs) and constituting a robust model of the human blood-brain barrier [109]. Otherwise, the iPSC cells can also generate primitive neural stem cells (NSCs), which can differentiate into neurons, astrocytes, or oligodendrocytes [110]. These BAGV sequences data obtained in our study could be used not only in future viral studies, but also in development of reverse genetic reagents or reliable diagnostic tools for investigation of this virus in human populations.

## Figures and Tables

**Table 1:** Description of primers used for full-genome characterization of Bagaza virus isolates.

Primer	Sequence 5′-3′	Direction	Position on Genome	Melting Temperature (°C)
5′raceBAGV1	CATCAATCCGACATCCAGAG	Antisense	Envelope	53
5′raceBAGV2	CCTTTCGGAAGCTTTTCAAG	Antisense	Envelope	53
BAG28F	TTGACAGCTCAACACAAGTGC	Sense	Envelope	55
BAG1037R	CCATCACGACATCAATCCAC	Antisense	Envelope	55
BAG572F	GCTCTGGATGTCGGATTGAT	Sense	Envelope	55
BAG2069R	TTGTCCCCGATGATGATGTA	Antisense	NS1	55
BAG3SEG1F	TCATTTCGAGTTGGCTGTGT	Sense	NS1	55
BAG3SEG1R	TATTGGACATGGGTGGAGTG	Antisense	NS2B	55
BAG3SEG2F	GTGTAAGGTCCGTGGGAAGA	Sense	NS2A	55
BAG3SEG2R	CAAACCAATCAGCACTCCAC	Antisense	NS3	55
BAG3538F	GAACCATTTCAGCTGGGTGT	Sense	NS3	55
BAG5064R	CCGACAAGAATGCCATTACC	Antisense	NS4A	55
BAG4825F	TCGTATGGAGGACCTTGGAA	Sense	NS3	55
BAG6324R	CCAAAGCTCAACTGGGTTGT	Antisense	NS4B	55
BAG6SEG1F	CGAGCCGGGTTATTGATAGT	Sense	NS4B	55
BAG6SEG1R	ACCTGCTGCTGTTCTCCTTT	Antisense	NS5	55
BAG6SEG2F	GACGTTTTTGACACCACTGC	Sense	NS5	55
BAG6SEG2R	GACGCGGTCTTCTACCATTT	Antisense	NS5	55
BAG8234F	GAAAGAACTGGAACGGATGC	Sense	NS5	55
BAG9750R	CTCGGGGATGTCTTTTCTGA	Antisense	NS5	55
BAG9329F	AGAATGGACCCAGAGCACAG	Sense	NS5	55
BAG10853R	TCCCAGGTGTCAATATGCTG	Antisense	NS5	55
3′raceBAGV1	AAAGCGCTCAATACCGACTC	Sense	NS5	53
3′raceBAGV2	AGTCAGGCCACAGGTTTTGT	Sense	NS5	53

BAGV: Bagaza virus.

**Table 2:** Description of Bagaza virus isolates used in this study.

Isolate	Origin	Year of Isolation	Specie	Accession Numbers
ArA23139	Dezidougou (CI)	1988	*Culex poicilipes*	MF380429
ArD54139	Dakar-Bango (SEN)	1989	*Culex poicilipes*	MF380430
ArUS20100	Matam (SEN)	1989	*Culex poicilipes*	MF380424
ArD99335	Barkedji (SEN)	1994	*Culex neavei*	MF380431
ArD137998	Diawara (SEN)	2000	*Culex poicilipes*	MF380425
ArD138018	Diawara (SEN)	2000	*Culex poicilipes*	MF380426
ArD152146	Diawara (SEN)	2001	*Culex poicilipes*	MF380433
ArD152207	Diawara (SEN)	2001	*Culex poicilipes*	MF380432
ArD171075	Barkedji (SEN)	2004	*Culex poicilipes*	MF380427
ArD171102	Barkedji (SEN)	2004	*Culex poicilipes*	MF380428
ArD260266	Barkedji (SEN)	2014	*Culex neavei*	MF380434

SEN: Senegal; CI: Côte d’Ivoire.

**Table 3:** Comparison of genomic regions between Bagaza virus isolates.

Genomic Regions	AY632545_DakAR B209_CAR_1966	HQ644143_Spain_H_2010	Isolates Sequenced in This Study
5′ UTR	94 nt	94 nt	94 nt
Capsid	122 aa	122 aa	122 aa
prM	177 aa	177 aa	177 aa
Envelope	501 aa	501 aa	501 aa
NS1	342 aa	342 aa	342 aa
NS2A	226 aa	226 aa	226 aa
NS2B	132 aa	132 aa	132 aa
NS3	619 aa	619 aa	619 aa
NS4A	126 aa	126 aa	126 aa
2K	23 aa	23 aa	23 aa
NS4B	253 aa	253 aa	253 aa
NS5	905 aa	905aa	905 aa
3′ UTR	566 nt	439 nt	576 nt
Total length	10,941 nt	10,794 nt	10,951 nt

nt: nucleotide; aa: amino acid. UTR: untranslated region; Gene lengths of reference sequence BAGV: AY632545 as described by Kuno, G.; Chang, G.J. 2007 [3].

**Table 4:** Location of highly conserved flavivirus amino acid motifs across Envelope, NS1, NS3 and NS5 proteins.

Gene	Amino Acid Motifs Previously Described in MBFVs ^#^	Cons * on Used NKVFs/ISFs Genome	Positions on BAGV Genome	Cons * on BAGV	Except on These BAGV Isolates	Replaced by This Consensus Sequence
**E**	DRGWGNGC	**YES**	387–394	**YES**	ARD171075_BARKEDJI_SEN_2004	**A**R**SR**GNGC
	GLFGKGS	only on NKVFs	395–401	**YES**	ARD171102_BARKEDJI_SEN_2004	GLF**A**KGS
	GHLKCRV	**NO** RBV (GH**VD**CRV) ModV (GHVSC**K**V)	573–579	**YES**		
	PFGDSYIV	NO	667–675	**NO**	All BAGV isolates	PFGDS**F**ILV
**NS1**	DTAWDFGS	NO	712–719	**YES**	ARD171102_BARKEDJI_SEN_2004	DPAWDFGS
					EU684972_96363_India_1996	DAAWDFGS
	GCWYGMEI	only on NKVFs	1118–1125	**YES**		
	YGMEIRP	YES	1120–1127	**YES**	ARD54139_DAKAR-BANGO_SEN_1989	YGMEIRT
**NS3**	GTSGSPI	YES	1633–1639	**YES**		
	GLYGNG	only on NKVFs and CxFV	1648–1653	**YES**	ARUS20100_MATAM_SEN_1989	G**V**YGNG
	LAPTRVV	YES	1722–1728	**YES**	ARD137998_DIAWARA_SEN_2000	LPLTRLV
					ARD138018_DIAWARA_SEN_2000	SPLTRLV
					ARD171075_BARKEDJI_SEN_2004	SAPTRLV
	DVMCHATF	Only on NKVFs	1759–1766	**NO**	ARD138018_DIAWARA_SEN_2000	DVMCHAPL
					Other BAGV isolates	DVMCHATL
	MDEAHF	YES	1784–1789	**YES**	ARD138018_DIAWARA_SEN_2000	MYEAHF
	SIAARGY	YES	1794–1800	**YES**		
	MTATPPG	YES	1815–1821	**YES**		
	ISEMGAN	YES	1911–1917	**YES**		
	SAAQRRGR	YES	1954–1961	**YES**		
**NS5**	DLGCGRG	YES	2601–2607	**YES**		
	SRNSTHEMY	YES	2734–2741	**NO**	ARD54139_DAKAR-BANGO_SEN_1989	WRNPNHEMY
					Other BAGV isolates	SRNSNHEMY
	NMMGKREKK	YES	2977–2986	**YES**		
	ADDTAGWDT	YES	3056–3064	**YES**		
	WMTTEDML	YES	3330–3337	**YES**		

Cons *: conservation of motif; YES: conserved motif; NO: non-conserved motif; BAGV: Bagaza virus; ISFs: insect-specific flaviviruses; Culex flavivirus (CxFV) and Aedes Flavivirus (AeFV); NKVFs: no-known vector flaviviruses ; Modoc virus (ModV) and Rio Bravo virus (RBV); **^#^**: Conserved amino acid motifs as described in mosquito-borne flaviviruses (MBVFs) by Kuno, G.; Chang, G.J. 2007 [3]; Positions with amino acid different on BAGV polyprotein are highlighted in black for conservative mutations and in red for non-conservative mutations; Isolates name is labeled in the following format: Identification number, Origin, country code (SEN: Senegal, CI: Côte d’Ivoire, CAR: Central African Republic), and year of isolation; Except for the Indian Isolate (accession number, name, country, and year of isolation).

**Table 5:** Episodes of positive diversifying selection on Bagaza virus proteins.

Proteins		Number of Sites Detected by Method				Evidence of Positive Selection
		SLAC (*p* < 0.1)	FUBAR (Posterior Probability ≥ 0.9)	MEME (*p* < 0.1)	Branch-Site REL (*p* < 0.05)	
Capsid	Sites under negative selection (dN/dS < 1)	0	4	0	0	YES
	Sites under positive selection (dN/dS > 1)	0	2	2	0	
prM	Sites under negative selection (dN/dS < 1)	6	11	0	0	NO
	Sites under positive selection (dN/dS > 1)	0	0	0	0	
E	Sites under negative selection (dN/dS < 1)	15	88	0	0	YES
	Sites under positive selection (dN/dS > 1)	0	1	15	4	
NS1	Sites under negative selection (dN/dS < 1)	7	33	0	0	YES
	Sites under positive selection (dN/dS > 1)	0	0	11	3	
NS2A	Sites under negative selection (dN/dS < 1)	3	9	0	0	YES
	Sites under positive selection (dN/dS > 1)	0	0	1	1	
NS2B	Sites under negative selection (dN/dS < 1)	4	8	0	0	NO
	Sites under positive selection (dN/dS > 1)	0	0	0	0	
NS3	Sites under negative selection (dN/dS < 1)	17	63	0	0	YES
	Sites under positive selection (dN/dS > 1)	0	0	4	1	
NS4A	Sites under negative selection (dN/dS < 1)	2	5	0	0	NO
	Sites under positive selection (dN/dS > 1)	0	0	0	0	
NS4B	Sites under negative selection (dN/dS < 1)	2	14	0	0	YES
	Sites under positive selection (dN/dS > 1)	0	0	3	1	
NS5	Sites under negative selection (dN/dS < 1)	0	274	0	0	YES
	Sites under positive selection (dN/dS > 1)	0	0	10	1	

Pervasive diversifying selection at posterior probability ≥ 0.9 with FUBAR model; Episodic diversifying selection at 0.1 significance level with SLAC and MEME models; Episodic diversifying selection at *p*-value *p* ≤ 0.05 with Branch-sites REL model.

## Supplementary Materials

The following are available online at http://www.mdpi.com/1999-4915/10/4/193/s1, Figure S1: Sequences of conserved structural RNA domains identified on the 3′ UTR of Bagaza genomes used in this study.
