# Supplementary material for: Full-Genome Characterization and Genetic Evolution of West African Isolates of Bagaza Virus
Source: Viruses. 2018 Apr 13;10(4):193. doi: 10.3390/v10040193 (PMC5923487; doi:10.3390/v10040193)
Supplement: Supplementary file 1 [file viruses-10-00193-s001.pdf]

```
>Consensus sequence RNA structure 1      Reverse reading direction    P= 0.671490
ACAAAACCUGUGGCCUGACUCUGGGUGCCUAUCCUCUCCAACGCCAGGGAGCUCUCCGAGUCGGUAUUGAGCGCUUUGCGUGUUGAGUGUCGGACACUUUGGUGACCCAGUCCUCCUGGG
.....(((.(((..(((((.((((((....))))....((((((((((.((((.(((...)))....)))).))))).)))))))).))))).)))...)))..(((((.....)))))

>Consensus sequence RNA structure 2      Reverse reading direction    P= 0.994641
GGCCACGGGGGCCUUACGGCUUUGUUUACCCAGUCCACCUGGGGCAUGUCUUUACUCAGGCAGCACUGCAUGCUAGUGGCACAAAACCUGUGGCCUGACUCUGGGUGCCUAUCCUCUCCA
((((((((((((....))))((((...(((((.....)))))((.((((((......)))))).((((((.....))))))))))))..))))))))).......((((....)))).....

>Consensus sequence RNA structure 3      Foward reading direction     P= 0.976295
GCCACUAGCAUGCAGUGCUGCCUGAGUAAAGACAUGCCCCAGGUGGACUGGGUAAACAAAGCCGUAAGGCCCCCGUGGCCCAAGCAAGGGGAUGGUGUGACCCUUGUGUAAGGACUAGAG
(((((....((.((((.(..((((.(((......)))..))))..))))).)).......(((....)))....))))) ((..(((((((..........)))))))....)).......

>Consensus sequence RNA structure 4      Reverse reading direction    P= 0.846482
GGGGUCUCCCUCUAACCUCUAGUCCUUACACAAGGGUCACACCAUCCCCUUGCUUGGGCCACGGGGGCCUUACGGCUUUGUUUACCCAGUCCACCUGGGGCAUGUCUUUACUCAGGCAGCA
((((...))))...((((..((((.....((((((..........))))))...))))...))))(((....)))........(((((.....)))))((.((((((......))))))).
```

LEGEND:
"." stands for an unpaired base, "(" corresponds to a paired base whose other base involved in the pair is further 3' and ")" corresponds to a paired base whose other base involved in the pair is further 5'.

Figure S1. Sequences of conserved structural RNA domains identified on the 3′ UTR of Bagaza genomes used in this study. Analysis was performed using the RNAz method implemented in the Vienna RNA Websuite (Gruber, et al.; 2008 [22]). A classification probability above 0.5 was considered as significant. "." stands for an unpaired base, "(" corresponds to a paired base whose other base involved in the pair is further 3′ and ")" corresponds to a paired base whose other base involved in the pair is further 5′.
